# Supplementary material for: A stabilized tandem antigen chimera that elicits potent malaria transmission-reducing activity
Source: Nat Commun. 2026 Jan 24;17:2010. doi: 10.1038/s41467-026-68761-1 (PMC12936105; doi:10.1038/s41467-026-68761-1)
Supplement: Supplementary file 1 — Supplementary Information [file 41467_2026_68761_MOESM1_ESM.pdf]

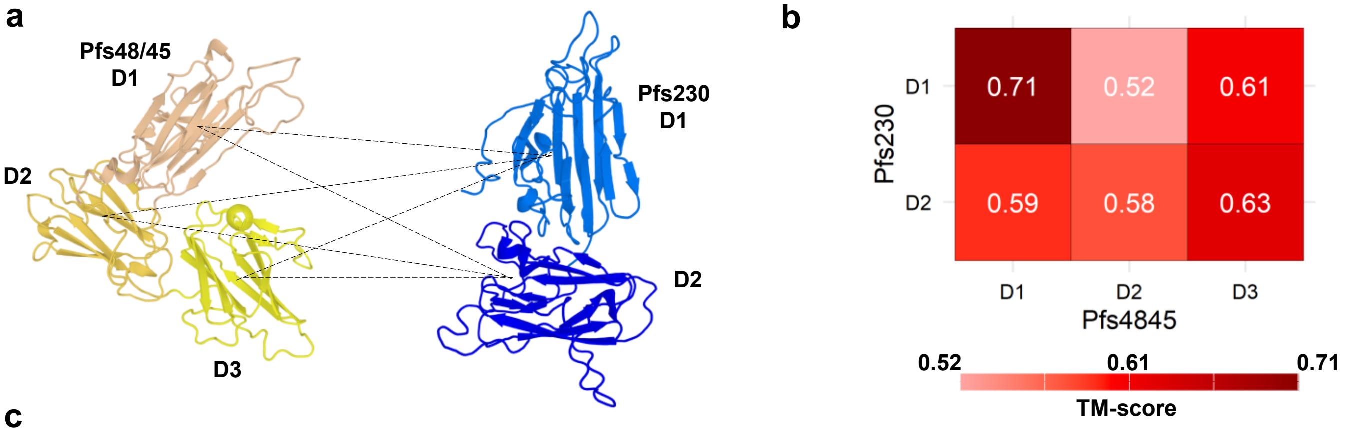

c

```
Pfs230-D2     732 NK-INGCAFLDEDEEEEKYGNQIEED--EHNEKIKMKTFFTQNIYKK-------NNIYP-CYMKLYSGDIGGILFPKNIKSTTCF
Pfs48/45-D3   292 KKVIHGCNFSSNVSSKYTFTDSLDISLVDDSAHISCNVHLSEPKYNHLVGLNCPGDIIPDCFFQVYQPE---------------S
                  :* *:** * .: ..: .: :.:: .  : . :*. :..:::  *::       .:* * *::::*. :

Pfs230-D2         EEMIPYNKEIKWNKENKSLGNLVNNSVVYNKEMNAKYFNVQYVHIPTSYKDTLNLFCSIILKEEESNLISTSYLVYVSINEELN 889
Pfs48/45-D3       EELEPSN--IVYLDSQINIGDIEYYEDAEGDDKIKLFLIVGSVPKTTS-------FTCICKKDKKSAYMTVTI-------DSAG 429
                  **: * *  * : ..: .:*::   . . ..:    :: *  * .**       * .*  *:::*  ::.:        :. .
```

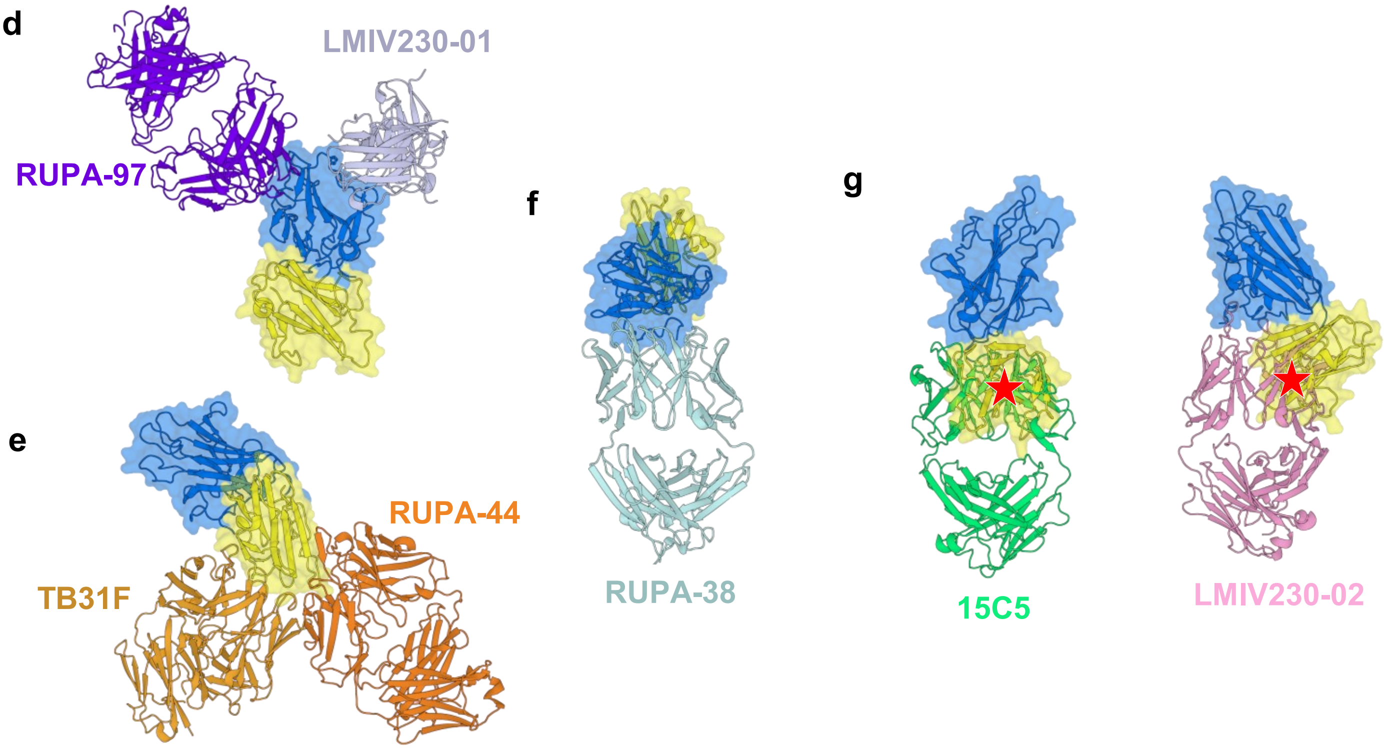

Supplementary figure 1. Design of a tandem antigen chimera to immunofocus Pfs230-D1 with Pfs48/45-D3 emulating epitope masking by Pfs230-D2. **a** Domain cross-comparison of Pfs230-D1D2 and Pfs48/45-D1D2D3 (PDBs 7USS and 7ZXF) and **b** heatmap indicating template modeling (TM)-scores for each domain-domain alignment. **c** Amino acid sequence alignment of Pfs230-D2 and Pfs48/45-D3. **d-e** Design-1 model with superimposed potent anti-Pfs230 (RUPA-97 and LMIV230-01) or anti-Pfs48/45 (TB31F and RUPA-44) mAb structures (PDBs 7UVQ, 7JUM, 6E63, and 7UXL) **f-g** Design-1 model with superimposed nonfunctional anti-Pfs230 mAb structures (RUPA-38, 15C5 and LMIV230-02; PDBs 7UVO, 7UVQ, and 7UVS).

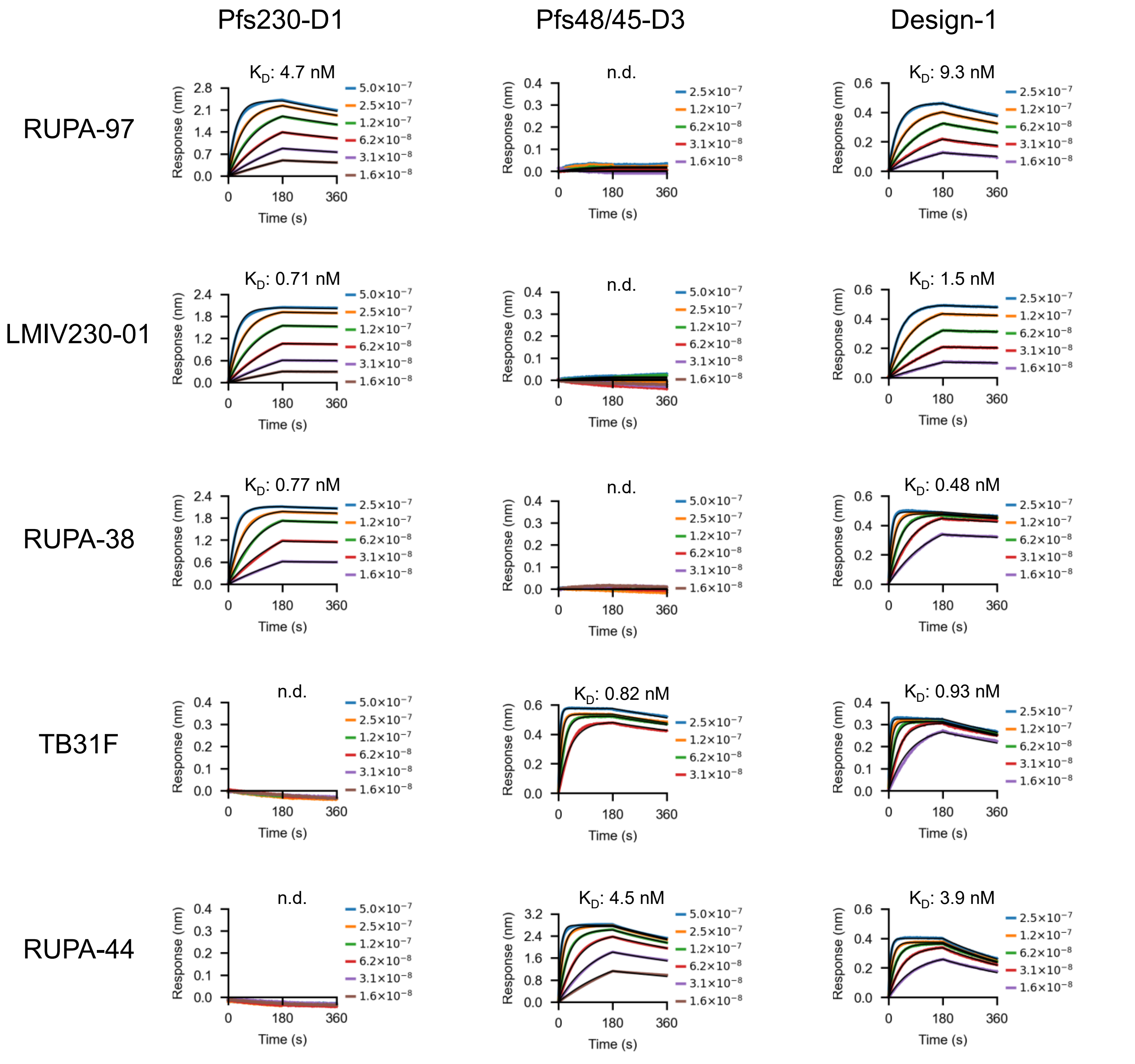

Supplementary figure 2. Biolayer interferometry data for Pfs230-D1, Pfs48/45-D3, and Design-1 with specified Fabs. Values for all calculated kinetic parameters corresponding to these plots can be found in Supplementary table 1. Binding affinities that could not be determined (n.d.) are indicated. Analyte concentrations (M) are shown.

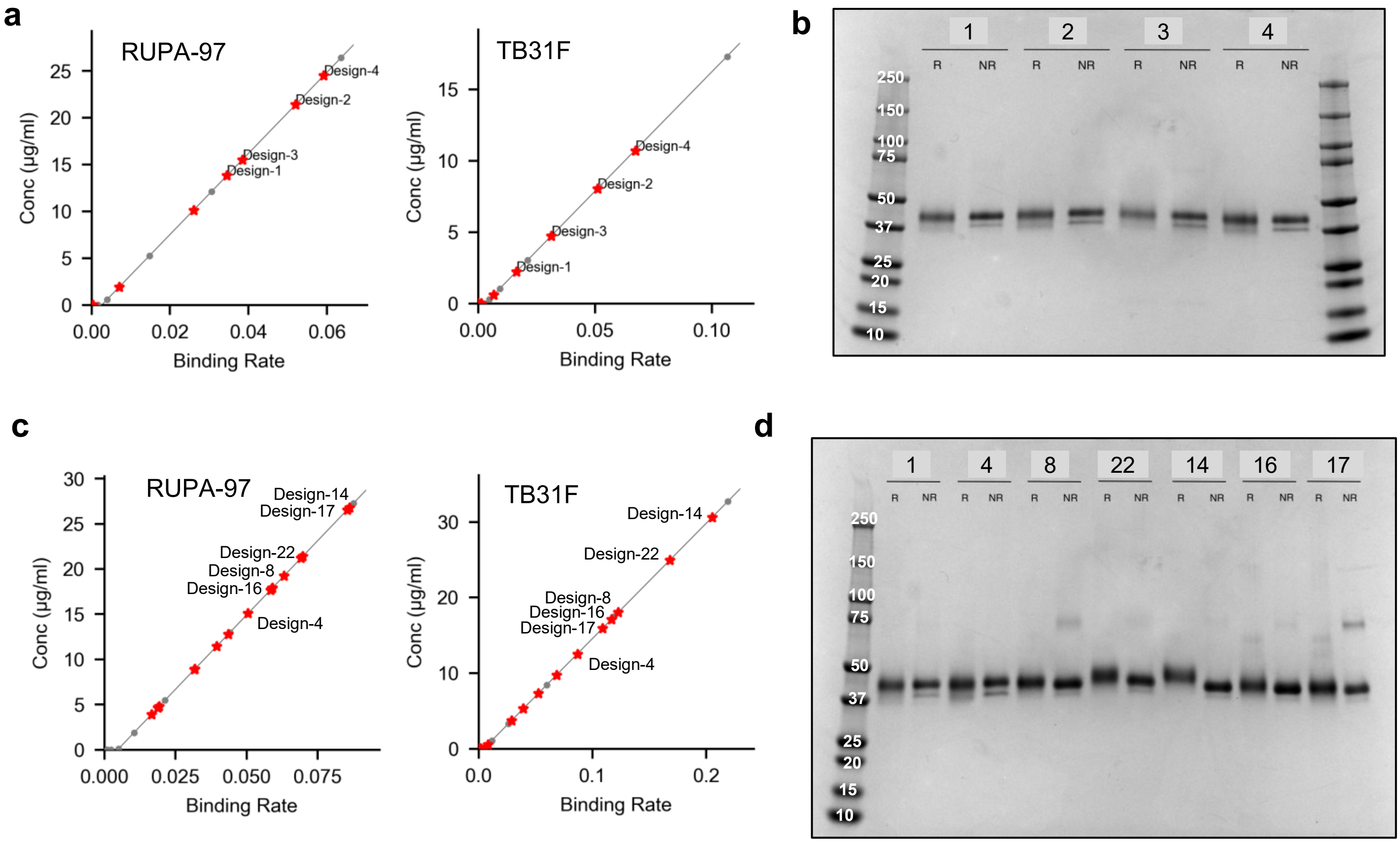

Supplementary figure 3. Antigen series expressions and purifications. **a** Processed BLI quantitation assay data for supernatants containing Designs 1 to 7. **b** SDS-PAGE analysis under reducing conditions (R) and non-reduced conditions (NR) using β-mercaptoethanol for peaks reported in **Figure 2d**. **c** Processed BLI quantitation assay data for supernatants containing Designs 8 to 22. **d** SDS-PAGE analysis under reducing conditions (R) and non-reduced conditions (NR) using β-mercaptoethanol for peaks reported in **Figure 2i**.

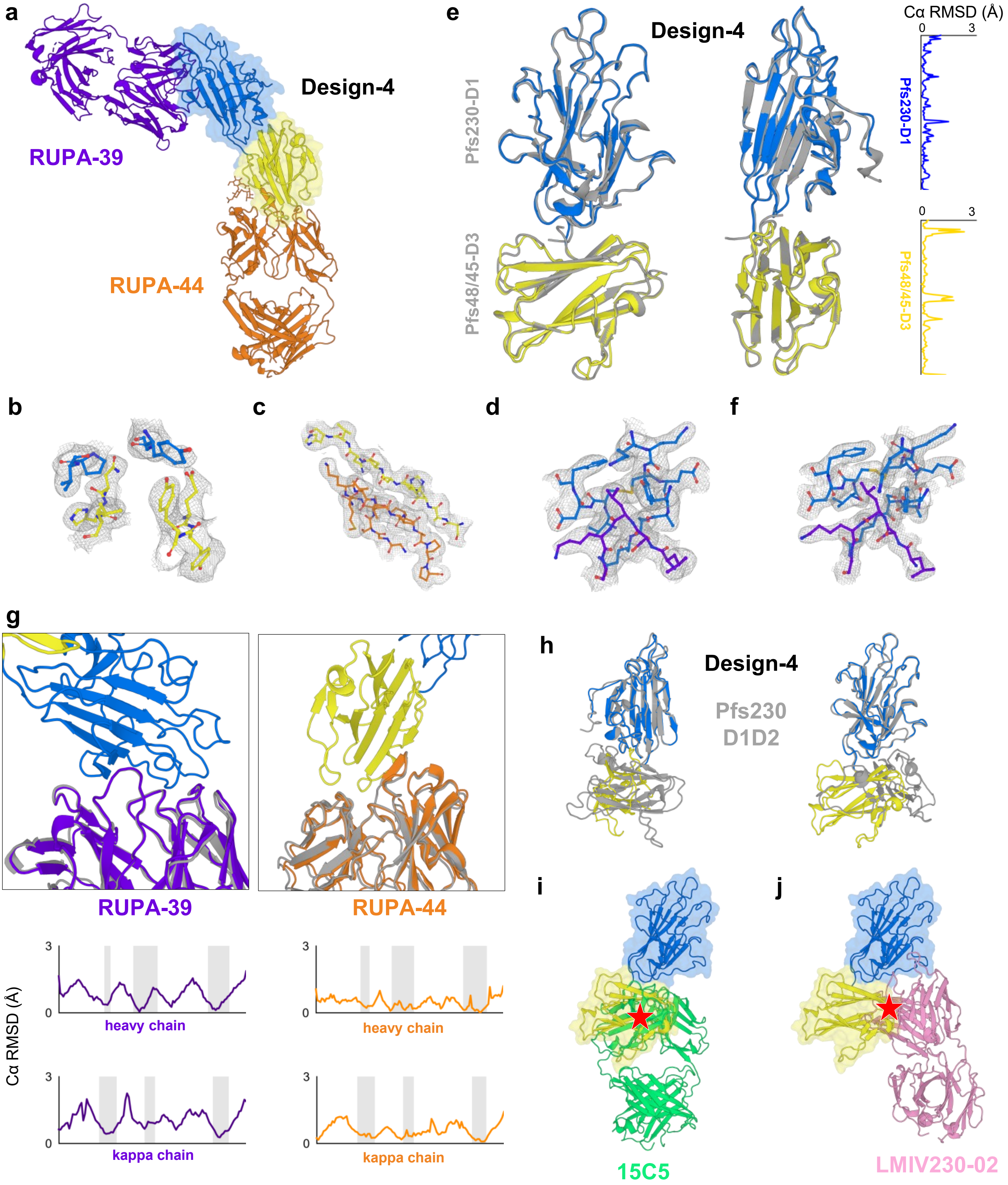

Supplementary figure 4. Structural comparisons of Design-4. **a** Crystal structure of Design-4 bound by RUPA-39 and RUPA-44 Fabs with composite omit map contoured at 1.0 sigma at the interfaces of **b** Pfs230:Pfs48/45 domain, **c** Design-4:RUPA-44, and **d** Design-4:RUPA-39. **e** Design-4 crystal structure superimposed with Pfs230-D1 and Pfs48/45-D3 crystal structures (PDB 7UXL and 9N8I). **f** Composite omit map contoured at 1.0 sigma at the interface of Pfs230-D1:RUPA-39 crystal structure. **g** Design-4 crystal structure superimposed with Fab-bound crystal structures (PDB 7UXL and 9N8I). Cα RMSD values are shown for each alignment; CDR residues (Kabat boundaries) are indicated with shading. **h** Pfs230-D1D2 crystal structure (PDB 7USS), and **i-j** with nonfunctional mAb structures (PDB 7UVQ and 7UVS). Grey colouring indicates previously reported structures when overlayed for comparison. Red stars indicate regions of steric clashing between antibody and antigen.

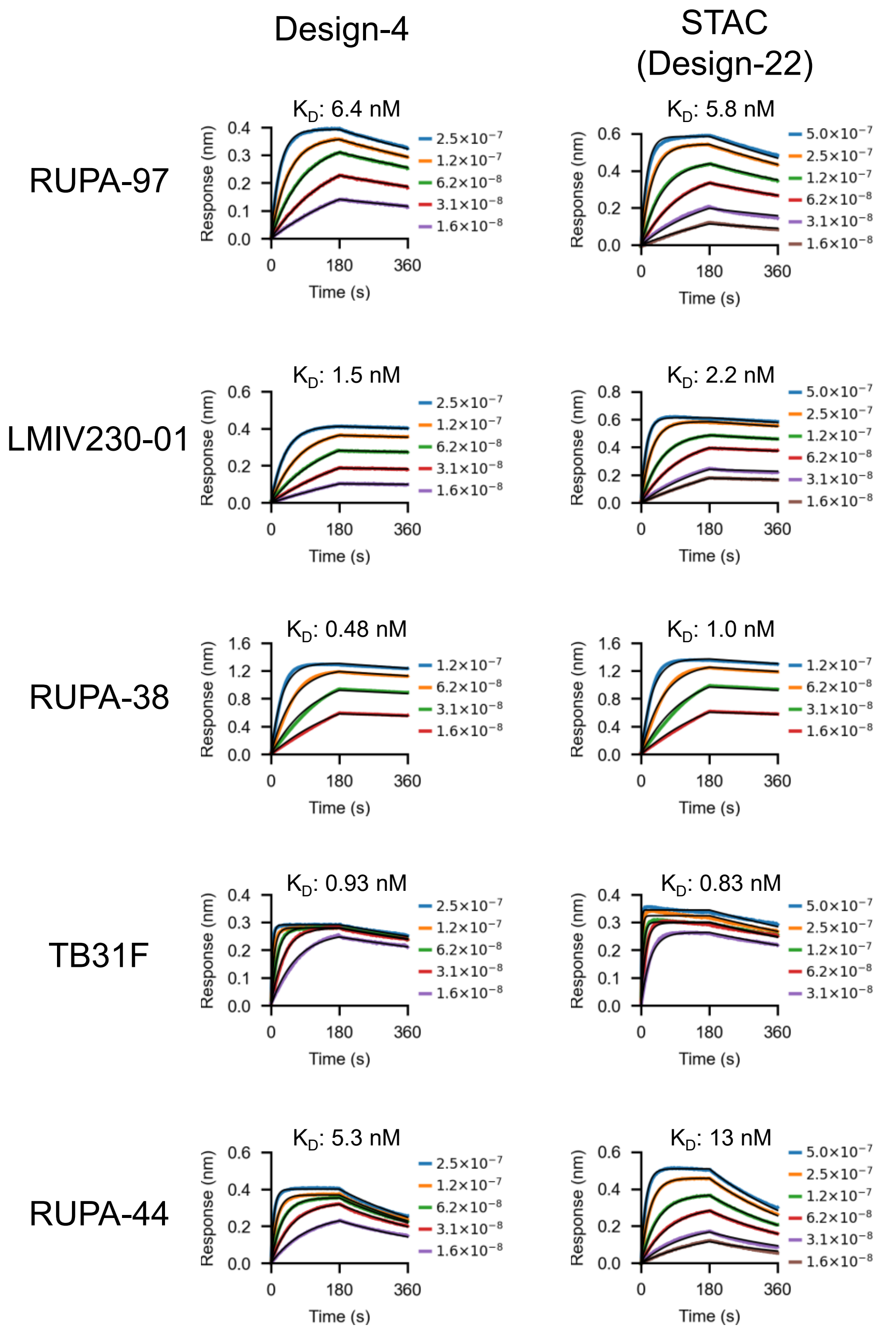

Supplementary figure 5. Biolayer interferometry data for Design-4 and Design-22 with specified Fabs. Analyte concentrations (M) are shown. Values for all calculated kinetic parameters corresponding to these plots can be found in **Supplementary table 1**.

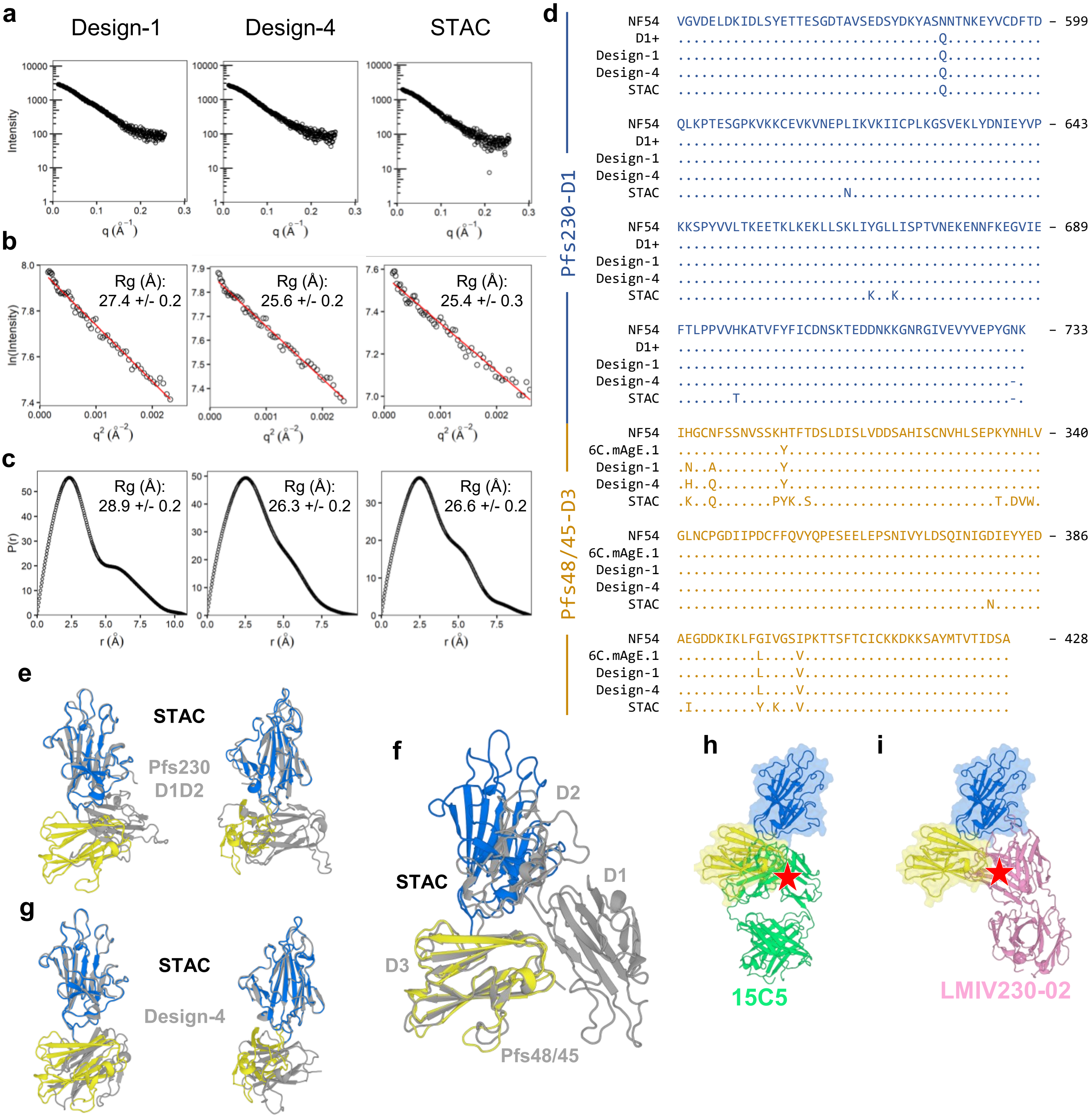

Supplementary figure 6. Structural details of STAC. **a-c** SAXS scattering plots, Guinier plots, and Distance distribution P(r) plots for Design-1, Design-4, and STAC. **d** Amino acid sequences of Pfs230-D1 and Pfs48/45-D3 (NF54 sequences) with mutations indicated for designed antigens. Design-22 cryo-EM structure superimposed with **e** Pfs230-D1D2 crystal structure (PDB 7USS), **f** Pfs48/45-D1D2D3 crystal structure (PDB 7ZXF), and **g** Design-4 crystal structure. **h-i** Design-22 cryo-EM structure with superimposed structures of non-functional mAbs (PDB 7UVQ and 7UVS). Grey colouring indicates previously reported structures when overlayed for comparison. Red stars indicate regions of steric clashing between antibody and antigen.

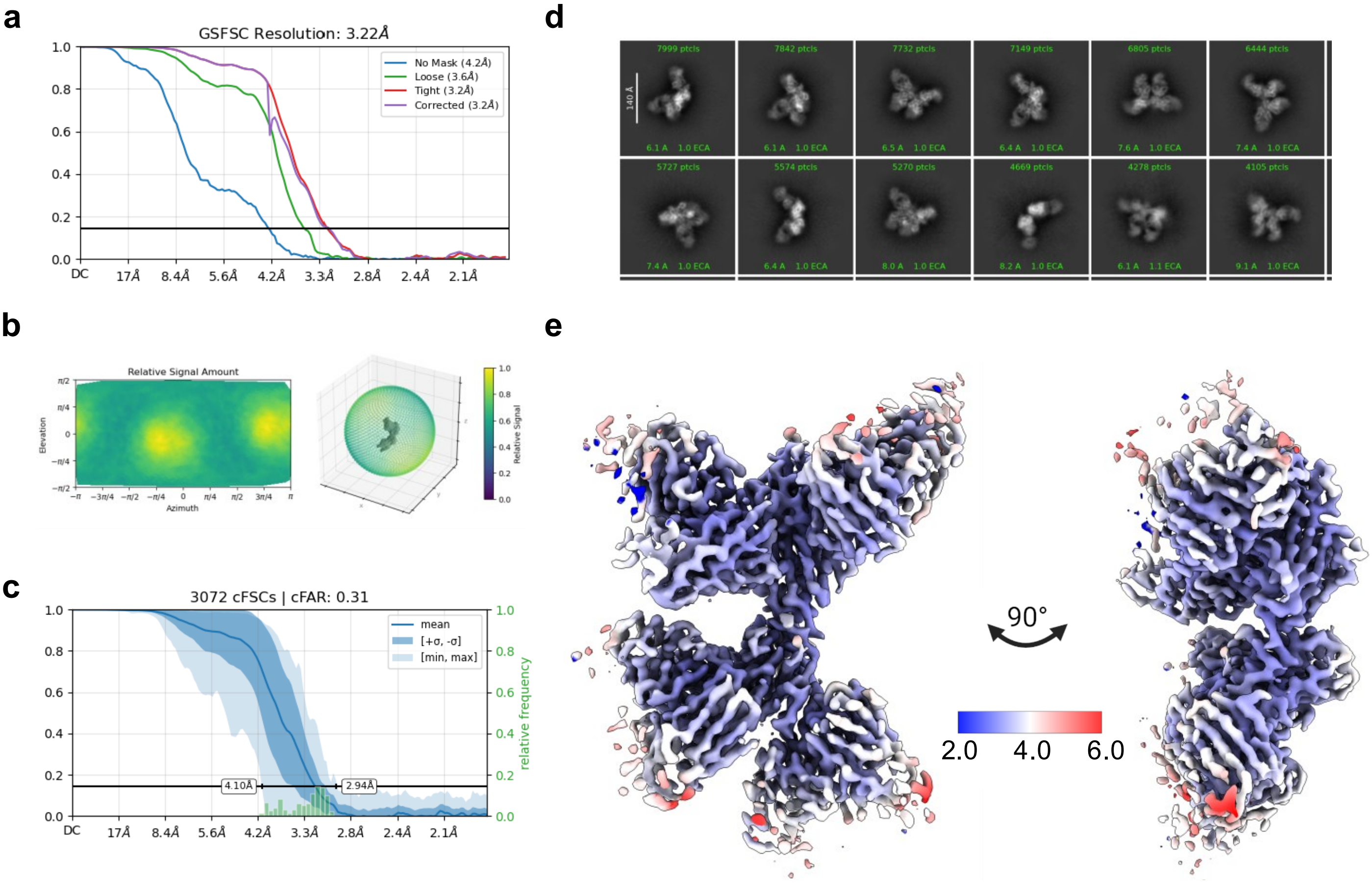

Supplementary figure 7. Cryo-EM map validation for the structure of the stabilized tandem antigen chimera (STAC) of Pfs230 and Pfs48/45 bound by four potent mAbs. Shown are **a** the Fourier shell correlation curves following the gold-standard method with correction for the effects of masking, **b** the particle distribution, **c** the cFAR plot and score, **d** representative 2D classes, and **e** the cryo-EM map coloured by local resolution for this locally refined map.

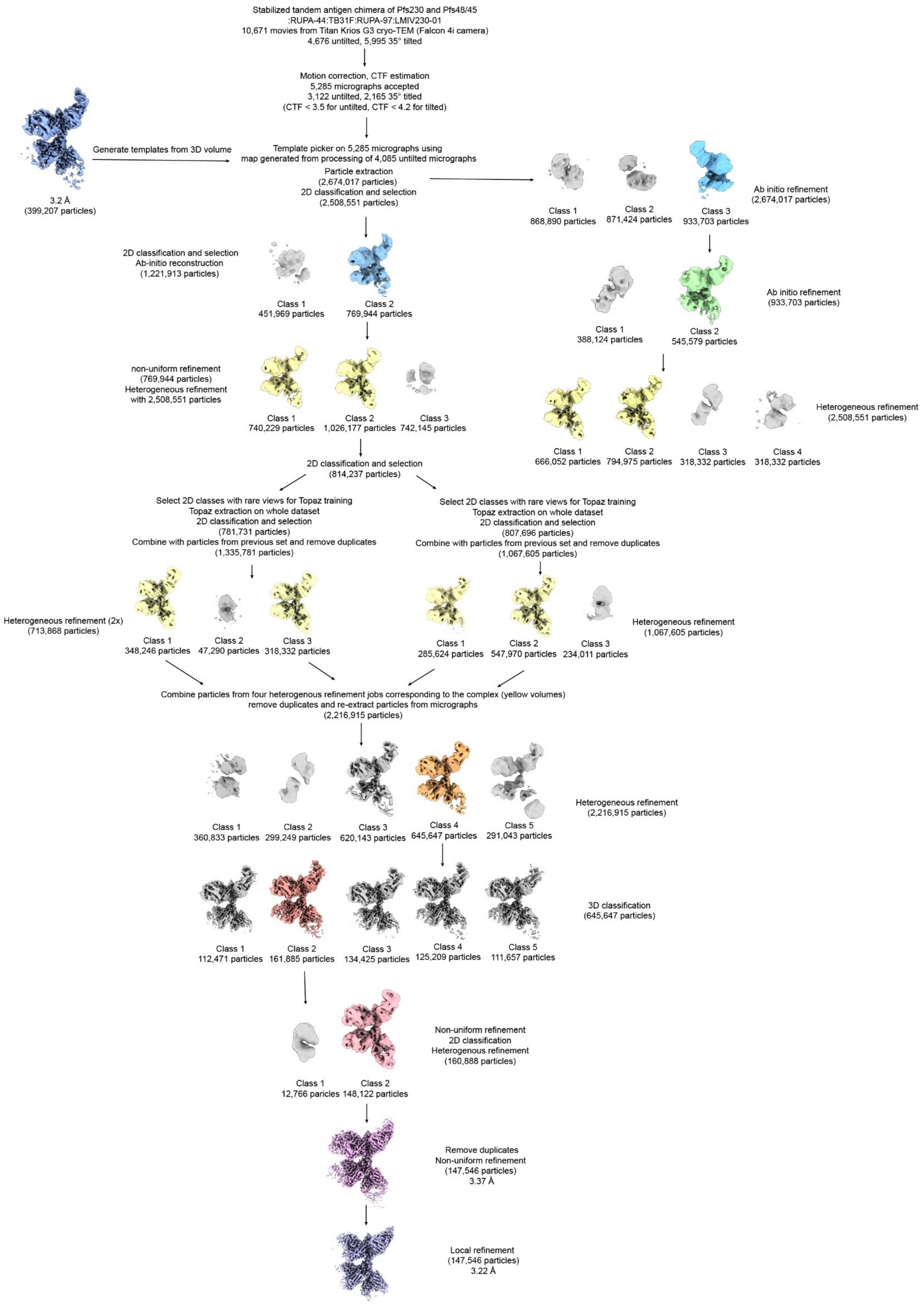

Supplementary figure 8. Cryo-EM data processing workflow.

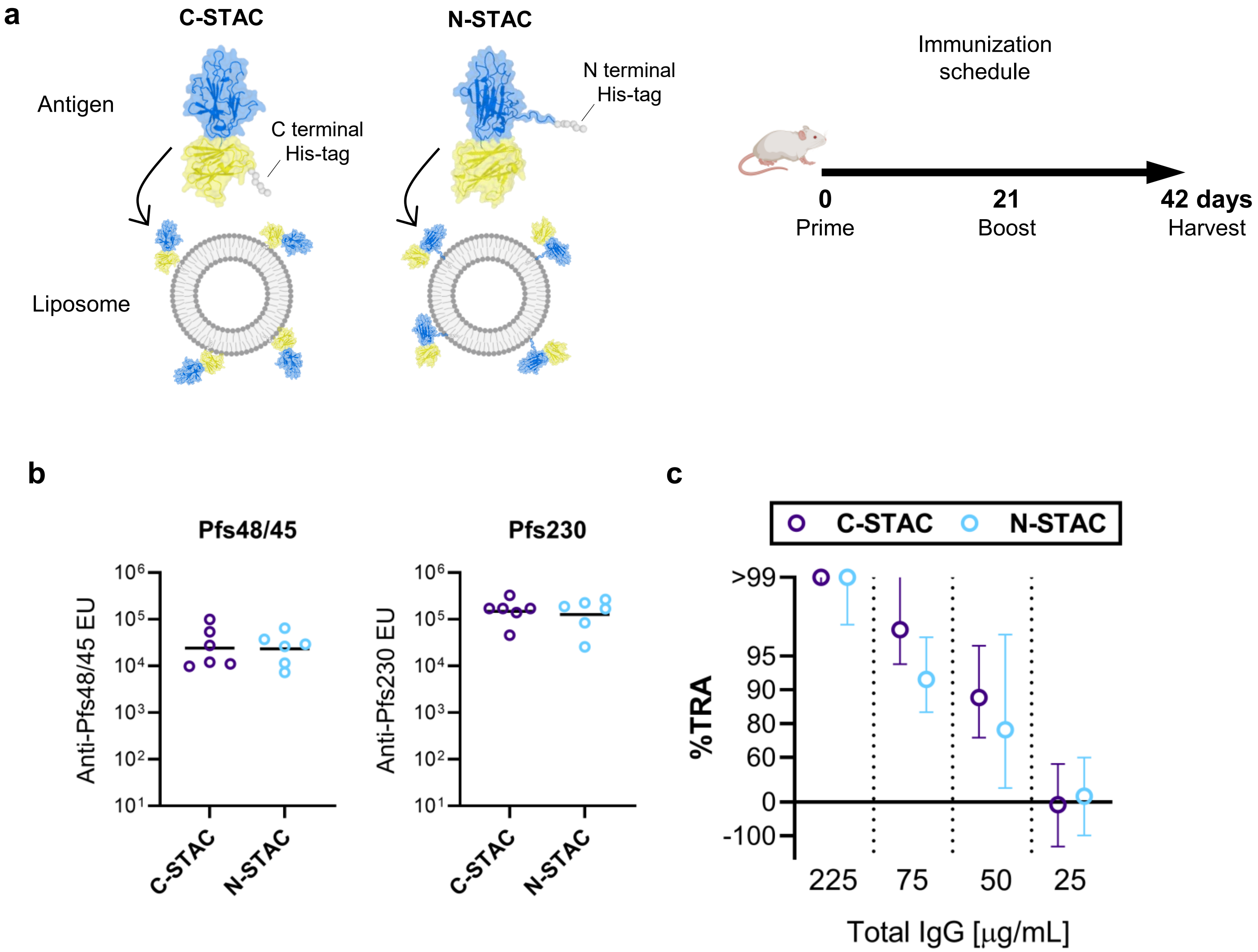

Supplementary figure 9. Antigen orientation does not impact immunogenicity on CPQ liposomes. **a** Mouse immunization scheme for STAC antigens with N and C terminal Histidine tags on CPQ liposomes (created with Biorender.com). The antigen groups received 1 μg dose by intramuscular injection in a prime-boost regimen **b-c** ELISA and SMFA titrations for head-to-head comparison of N- and C- terminally His tagged STAC. The SMFA data at 75 μg/ml came from two feeds, while all other data was generated from a single feed. There was an insignificant difference in ELISA titers ($p>0.74$ for both panels by a student t-test using log-transformed values) or functional activity ($p=0.31$ by a linear regression model).

a

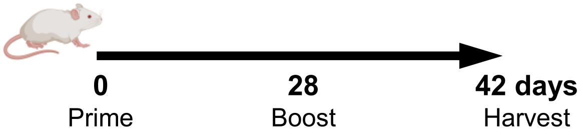

b

Platform	Antigen	Antigen dose		Immunogen dose	
		pmol	μg	pmol	μg
H. pylori ferritin	STAC	156	5.53	6.51	9.12
	Pfs230-D1 + Pfs48/45-D3 cocktail	313	5.68	13.0	12.4
	Pfs230-D1	156	3.18	6.51	6.56
	Pfs48/45-D3 (6C.mAgE2)	156	2.50	6.51	5.87
I53-50	STAC	156	5.53	2.60	12.0
	Pfs230-D1 + Pfs48/45-D3 cocktail	313	5.68	5.21	18.6
	Pfs230-D1 + Pfs48/45-D3 mosaic	313	5.68	5.21	18.6
	Pfs230-D1	156	3.18	2.60	9.66
	Pfs48/45-D3 (6C.mAgE2)	156	2.50	2.60	8.98
I53-50	no antigen control	0	0	2.60	6.45

c

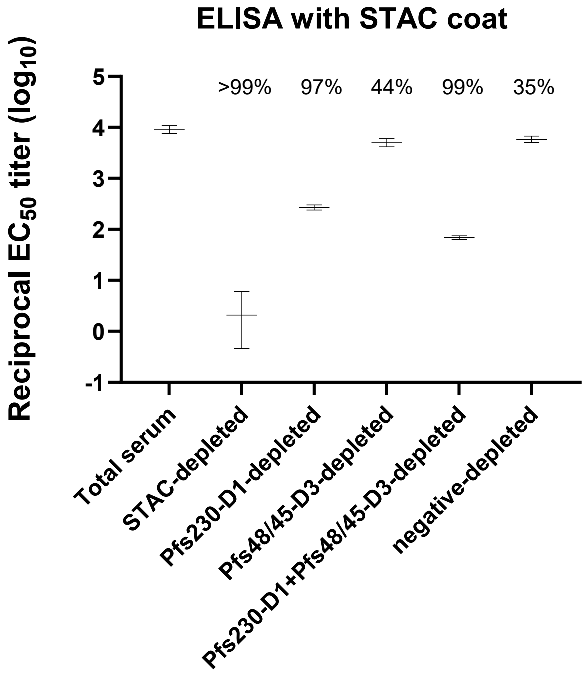

d

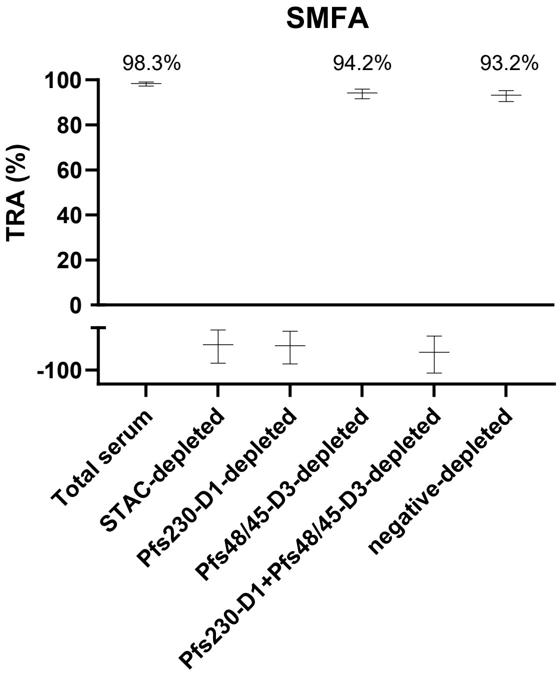

Supplementary figure 10. Protein nanoparticle immunogens presenting TBV antigens. Mouse immunizations with **a** immunization regimen (created with Biorender.com) and **b** dosages. **c** Reactogenicity and **d** functional activity of mouse sera from immunization with STAC on *H. pylori* ferritin depleted with specific antigens. **c** ELISAs demonstrating residual STAC-reactive sera after depletion with indicated antigens. Percentages indicate reduction in titers compared to those in total serum. **d** Transmission-reducing activity of the same depleted sera. TRA values are estimates from two independent experiments. All sera were tested at 1:81 dilution, except for STAC-depleted sera, which was tested at 1:27 dilution.

Supplementary table 1. Kinetic parameters and statistics for all BLI data reported in this study.

Fab	Antigen	K_D (M)	K_D Error	k_{on} (1/Ms)	k_{on} Error	k_{off} (1/s)	k_{off} Error	R^2
15C5	Pfs230-D1	2.94E-08	1.23E-10	9.27E+04	3.03E+02	2.72E-03	7.14E-06	0.9991
LMIV230-01	Pfs230-D1	7.07E-10	5.60E-11	7.58E+04	1.68E+02	5.36E-05	4.24E-06	0.9996
LMIV230-02	Pfs230-D1	5.15E-10	1.28E-11	3.82E+05	1.01E+03	1.97E-04	4.86E-06	0.9987
RUPA-38	Pfs230-D1	7.72E-10	3.35E-11	1.69E+05	4.96E+02	1.30E-04	5.65E-06	0.9992
RUPA-44	Pfs230-D1	n.d.		n.d.		n.d.		
RUPA-97	Pfs230-D1	4.70E-09	3.74E-11	1.47E+05	4.00E+02	6.91E-04	5.17E-06	0.9992
TB31F	Pfs230-D1	n.d.		n.d.		n.d.		
15C5	Pfs230-D1D2	n.d.		n.d.		n.d.		
LMIV230-01	Pfs230-D1D2	5.37E-10	1.35E-11	1.24E+05	1.07E+02	6.64E-05	1.66E-06	0.9999
LMIV230-02	Pfs230-D1D2	n.d.		n.d.		n.d.		
RUPA-38	Pfs230-D1D2	5.51E-10	1.58E-11	3.48E+05	1.03E+03	1.92E-04	5.47E-06	0.9984
RUPA-97	Pfs230-D1D2	4.94E-09	1.06E-11	2.20E+05	2.27E+02	1.09E-03	2.05E-06	0.9999
15C5	Pfs48/45-D3	n.d.		n.d.		n.d.		
LMIV230-01	Pfs48/45-D3	n.d.		n.d.		n.d.		
LMIV230-02	Pfs48/45-D3	n.d.		n.d.		n.d.		
RUPA-38	Pfs48/45-D3	n.d.		n.d.		n.d.		
RUPA-44	Pfs48/45-D3	4.50E-09	2.32E-11	2.37E+05	6.10E+02	1.07E-03	4.78E-06	0.999
RUPA-97	Pfs48/45-D3	n.d.		n.d.		n.d.		
TB31F	Pfs48/45-D3	8.19E-10	9.41E-12	8.03E+05	3.29E+03	6.57E-04	7.06E-06	0.9936
15C5	Design-1	n.d.		n.d.		n.d.		
LMIV230-01	Design-1	1.48E-09	2.92E-11	1.04E+05	1.67E+02	1.54E-04	3.03E-06	0.9998
LMIV230-02	Design-1	2.93E-09	1.07E-10	9.74E+04	5.30E+02	2.85E-04	1.03E-05	0.9970
RUPA-38	Design-1	7.24E-10	1.32E-11	4.43E+05	1.40E+03	3.21E-04	5.78E-06	0.9976
RUPA-44	Design-1	3.94E-09	1.30E-11	6.07E+05	1.54E+03	2.39E-03	5.07E-06	0.9986
RUPA-97	Design-1	9.34E-09	5.19E-11	1.25E+05	3.54E+02	1.17E-03	5.61E-06	0.9993
TB31F	Design-1	1.33E-09	1.39E-11	8.58E+05	4.78E+03	1.14E-03	1.01E-05	0.9888
15C5	Design-4	n.d.		n.d.		n.d.		
LMIV230-01	Design-4	1.47E-09	2.77E-11	1.09E+05	1.73E+02	1.59E-04	2.99E-06	0.9998
LMIV230-02	Design-4	1.25E-08	4.79E-10	2.94E+04	2.49E+02	3.67E-04	1.37E-05	0.9969
RUPA-38	Design-4	4.78E-10	1.09E-11	4.38E+05	1.14E+03	2.10E-04	4.75E-06	0.9984
RUPA-44	Design-4	5.34E-09	2.00E-11	4.98E+05	1.47E+03	2.66E-03	6.11E-06	0.9983
RUPA-97	Design-4	6.43E-09	2.36E-11	1.69E+05	2.97E+02	1.09E-03	3.49E-06	0.9997
TB31F	Design-4	9.26E-10	6.65E-12	9.27E+05	2.89E+03	8.58E-04	5.55E-06	0.9959
15C5	Design-22	n.d.		n.d.		n.d.		
LMIV230-01	Design-22	2.23E-09	2.83E-11	1.46E+05	3.16E+02	3.25E-04	4.06E-06	0.9995
LMIV230-02	Design-22	n.d.		n.d.		n.d.		
RUPA-38	Design-22	1.01E-09	4.67E-11	2.76E+05	1.77E+03	2.78E-04	1.27E-05	0.9959
RUPA-44	Design-22	1.28E-08	4.32E-11	2.34E+05	6.57E+02	3.01E-03	5.58E-06	0.9994
RUPA-97	Design-22	5.84E-09	4.49E-11	1.94E+05	4.90E+02	1.14E-03	8.23E-06	0.9982
TB31F	Design-22	8.28E-10	9.20E-12	1.29E+06	8.88E+03	1.07E-03	9.27E-06	0.9874

Supplementary table 2. X-ray crystallography data collection and refinement statistics.

	**Design-4 with RUPA-39 and RUPA-44 Fabs**	**Pfs230D1+ with RUPA-39 Fab and anti-kappa VHH**
**PDB ID**	9N8N	9N8I
**Beamline**	APS 23-ID-D	CLS CMCF-ID
**Wavelength (Å)**	1.03320	1.03322
**Space group**	C2	P 2_1
**Cell dimensions**		
**a,b,c (Å)**	227.99, 80.49, 137.73	62.95, 76.11, 88.64
**α, β, γ (°)**	90.0, 111.5, 90.0	90.0 96.4, 90.0
**Resolution (Å)**	75.26 - 2.22<br>(2.45 - 2.22)	48.54 - 1.85 (1.89-1.85)
**Total reflections**	1,309,963 (64,168)	525,836 (30,797)
**Unique reflections**	81,183 (4058)	70,596 (4309)
**Multiplicity**	16.1 (15.8)	7.4 (7.1)
**R_{meas}**	0.203 (2.001)	0.143 (1.647)
**R_{pim}**	0.051 (0.499)	0.071 (0.847)
**$\langle I/\sigma I \rangle$**	12.2 (1.7)	7.8 (1.5)
**$CC_{1/2}$**	0.998 (0.637)	0.995 (0.649)
**Spherical completeness (%)**	70.5 (13.6)	99.4 (98.8)
**Ellipsoidal completeness (%)**	93.6 (61.2)	-
**Refinement Statistics**		
**R_{work}/R_{free} (%)**	17.6/21.1	18.9/23.4
**Unique reflections for R_{free}**	2005	2005
**Molecules in ASU**	1	1
**Non-H atoms**	9657	6088
**Macromolecule**	9146	5749
**Water**	469	328
**Heteroatom**	42	11
**RMSD bonds (Å)**	0.0107	0.0088
**RMSD angles (°)**	1.24	1.09
**Ramachandran statistics**		
**Favored (%)**	97.1	97.3
**Allowed (%)**	2.9	2.7
**Outliers (%)**	0.0	0.0
**B-factors ($Å^2$)**		
**Wilson B-value**	39.6	32.9
**Average B-factors**	56.0	50.0

Supplementary table 3. Cryo-EM data collection and refinement statistics.

	STAC in complex with RUPA-97, LMIV230-01, RUPA-44, and TB31F Fabs
Accession codes	
PDB	9N8J
EMDB	EMD-49130
Data collection and processing	
Nominal magnification	130,000
Voltage (kV)	300
Total exposure (e^- /Å^2)	50-53.7
Defocus range (μm)	0.7-2.3
Calibrated physical pixel size (Å)	0.93
Symmetry imposed	C_1
Initial particle images (no.)	2,674,017
Final particle images (no.)	147,546
Map resolution (Å)	3.2
FSC threshold	0.143
Map resolution range (Å)	2.9 - 11.7
Model Building	
Initial models used (PDB code)	6E63, 7UXL, 7UVQ, 7UFW
Model resolution (Å)	3.2
FSC threshold	0.143
Model composition	
Non-hydrogen atoms	9,512
Number of residues built	1,226
Ligands	2
B factors (Å^2)	
Protein	71.8
Ligand	89.3
RMSD	
Bonds (Å)	0.004
Angles (°)	0.624
Validation	
Clashscore	4.7

Supplementary table 4. SAXS data collection parameters.

Source instrument	Anton Paar SAXSpace instrument
Wavelength (Å)	1.5418 Å
Beam geometry (sample-to-detector distance)	317.077 mm
Experimental setup	point collimation
q-measurement range ($Å^{-1}$)	0.0124 $Å^{-1}$ – 0.255 $Å^{-1}$
Exposure time	10 minutes
Number of exposures	6
Sample temperature	298.2 K
