# Supplementary material for: A stabilized tandem antigen chimera that elicits potent malaria transmission-reducing activity
Source: Nat Commun. 2026 Jan 24;17:2010. doi: 10.1038/s41467-026-68761-1 (PMC12936105; doi:10.1038/s41467-026-68761-1)
Supplement: Supplementary file 3 — Reporting Summary [file 41467_2026_68761_MOESM3_ESM.pdf]

nature portfolio

Corresponding author(s): Jean-Philippe Julien

Last updated by author(s): Dec 16, 2025

Reporting Summary

Nature Portfolio wishes to improve the reproducibility of the work that we publish. This form provides structure for consistency and transparency in reporting. For further information on Nature Portfolio policies, see our Editorial Policies and the Editorial Policy Checklist.

Statistics

For all statistical analyses, confirm that the following items are present in the figure legend, table legend, main text, or Methods section.

n/a	Confirmed	
☐	☒	The exact sample size (*n*) for each experimental group/condition, given as a discrete number and unit of measurement
☐	☒	A statement on whether measurements were taken from distinct samples or whether the same sample was measured repeatedly
☒	☐	The statistical test(s) used AND whether they are one- or two-sided<br>*Only common tests should be described solely by name; describe more complex techniques in the Methods section.*
☒	☐	A description of all covariates tested
☒	☐	A description of any assumptions or corrections, such as tests of normality and adjustment for multiple comparisons
☐	☒	A full description of the statistical parameters including central tendency (e.g. means) or other basic estimates (e.g. regression coefficient) AND variation (e.g. standard deviation) or associated estimates of uncertainty (e.g. confidence intervals)
☐	☒	For null hypothesis testing, the test statistic (e.g. *F*, *t*, *r*) with confidence intervals, effect sizes, degrees of freedom and *P* value noted<br>*Give P values as exact values whenever suitable.*
☒	☐	For Bayesian analysis, information on the choice of priors and Markov chain Monte Carlo settings
☒	☐	For hierarchical and complex designs, identification of the appropriate level for tests and full reporting of outcomes
☒	☐	Estimates of effect sizes (e.g. Cohen's *d*, Pearson's *r*), indicating how they were calculated

Our web collection on statistics for biologists contains articles on many of the points above.

Software and code

Policy information about availability of computer code

Data collection	Data collection software used are described in the methods section and are commercially available or openly accessible. Software used includes: EPU 2 (ThermoFisher Scientific), ForteBio 8.2.0.7 (Sartorius), SAXSDrive version 2 (Anton Paar)

Data analysis	Data analysis was performed as described in the methods section using commercially available or openly accessible software. Software used includes: R version 4.1.2, Python version 3.7, GraphPad Prism 10.0.0 (GraphPad Software, Boston, Massachusetts USA), ForteBio 8.2.0.7 (Sartorius), cryoSPARC v3 (Structura Biotechnology), phenix.real_space_refine (Afonine, P. V. et al. Real-space refinement in PHENIX for cryo-EM and crystallography. Acta Crystallogr. Sect. Struct. Biol. 74, 531–544 (2018)), MolProbity (Chen, V. B. et al. MolProbity: all-atom structure validation for macromolecular crystallography. Acta Crystallogr. D Biol. Crystallogr. 66, 12–21 (2010)), UCSF Chimera (Pettersen, E. F. et al. UCSF Chimera-a visualization system for exploratory research and analysis. J. Comput. Chem. 25, 1605–1612 (2004)), UCSF ChimeraX (Goddard, T. D. et al. UCSF ChimeraX: Meeting modern challenges in visualization and analysis. Protein Sci. Publ. Protein Soc. 27, 14–25 (2018)), Topaz (Bepler, T. et al. Positive-unlabeled convolutional neural networks for particle picking in cryo-electron micrographs. Nat. Methods 16, 1153–1160 (2019).), PDBePISA (Krissinel, E. & Henrick, K. Inference of macromolecular assemblies from crystalline state. J. Mol. Biol. 372, 774–797 (2007)), The PyMOL Molecular Graphics System v2.3.4 (Schrödinger), Phaser (McCoy, A. J. et al. Phaser crystallographic software. J. Appl. Crystallogr. 40, 658–674 (2007)), phenix.refine (Adams, P. D. et al. PHENIX: a comprehensive Python-based system for macromolecular structure solution. Acta Crystallogr. D Biol. Crystallogr. 66, 213–221 (2010)), Coot , (Emsley, P., Lohkamp, B., Scott, W. G. & Cowtan, K. Features and development of Coot. Acta Crystallogr. D Biol. Crystallogr. 66, 486–501 (2010)), SBGrid (SBGrid Consortium), ATSAS package (version 3.2.1 ; EMBL, Hamburg, Germany), ProteinMPNN – v1.0.1 (model v_48_020) (Dauparas, J. et al. Robust deep learning-based protein sequence design using ProteinMPNN. Science 378, 49–56 (2022).), and AlphaFold2 (Jumper, J. et al. Highly accurate protein structure prediction with AlphaFold. Nature 596, 583–589 (2021); Mirdita, M. et al. ColabFold: making protein folding accessible to all. Nat. Methods 19, 679–682 (2022).)

For manuscripts utilizing custom algorithms or software that are central to the research but not yet described in published literature, software must be made available to editors and reviewers. We strongly encourage code deposition in a community repository (e.g. GitHub). See the Nature Portfolio guidelines for submitting code & software for further information.

Data

Policy information about availability of data

All manuscripts must include a data availability statement. This statement should provide the following information, where applicable:

- Accession codes, unique identifiers, or web links for publicly available datasets
- A description of any restrictions on data availability
- For clinical datasets or third party data, please ensure that the statement adheres to our policy

X-ray crystallography and cryoEM structures are accessible from the Protein Data Bank and Electron Microscopy Data Bank under IDs: 9N8N, Design-4 with RUPA-39 and RUPA-44 Fabs; 9N8I, Pfs230D1+ with RUPA-39 Fab and anti-kappa VHH; and 9N8J and EMD-49130, STAC in complex with RUPA-97, LMIV230-01, RUPA-44, and TB31F Fabs. This paper does not report original code. Raw SMFA data is available in the source data. Any additional information required to reanalyze the data reported in this paper is available from the corresponding author upon request.

Research involving human participants, their data, or biological material

Policy information about studies with human participants or human data. See also policy information about sex, gender (identity/presentation), and sexual orientation and race, ethnicity and racism.

Reporting on sex and gender	N/A
Reporting on race, ethnicity, or other socially relevant groupings	N/A
Population characteristics	N/A
Recruitment	N/A
Ethics oversight	N/A

Note that full information on the approval of the study protocol must also be provided in the manuscript.

Field-specific reporting

Please select the one below that is the best fit for your research. If you are not sure, read the appropriate sections before making your selection.

[x] Life sciences [ ] Behavioural & social sciences [ ] Ecological, evolutionary & environmental sciences

For a reference copy of the document with all sections, see nature.com/documents/nr-reporting-summary-flat.pdf

Life sciences study design

All studies must disclose on these points even when the disclosure is negative.

Sample size	For SMFA experiments mosquito group size was chosen based on previous studies (Miura, K. et al. PLoS ONE 2013).
Data exclusions	No data were excluded from analysis.

Replication	All SMFA experiments were repeated three or more times, unless otherwise specified.
Randomization	No randomisation was conducted.
Blinding	No blinding was conducted.

Reporting for specific materials, systems and methods

We require information from authors about some types of materials, experimental systems and methods used in many studies. Here, indicate whether each material, system or method listed is relevant to your study. If you are not sure if a list item applies to your research, read the appropriate section before selecting a response.

Materials & experimental systems

n/a	Involved in the study
☐	☒ Antibodies
☐	☒ Eukaryotic cell lines
☒	☐ Palaeontology and archaeology
☐	☒ Animals and other organisms
☒	☐ Clinical data
☒	☐ Dual use research of concern
☒	☐ Plants

Methods

n/a	Involved in the study
☒	☐ ChIP-seq
☒	☐ Flow cytometry
☒	☐ MRI-based neuroimaging

Antibodies

Antibodies used	Monoclonal antibodies described in this publication are not commercially available. All were produced and validated as described in publications which are cited in the manuscript and were used at concentrations as described in the text.
Validation	All antibodies were previously validated through Western blotting and gametocyte staining in previous publications, as cited in the text.

Eukaryotic cell lines

Policy information about cell lines and Sex and Gender in Research

Cell line source(s)	HEK293 cells were used to produce recombinant proteins, antibodies and antibody fragments.
Authentication	HEK293 cells are commercial cell line and were validated by their commercial distributor (ThermoFisher Scientific).
Mycoplasma contamination	HEK293 cells were frequently tested for myscoplasma contamination and no contamination was detected.
Commonly misidentified lines (See ICLAC register)	None were used.

Animals and other research organisms

Policy information about studies involving animals; ARRIVE guidelines recommended for reporting animal research, and Sex and Gender in Research

Laboratory animals	Mosquitoes used for transmission-blocking assays were 4–6 days old female Anopheles stephensi (SDA 500).
Wild animals	N/A
Reporting on sex	All mosquitoes used were female. Mice for lipid nanoparticle immunizations were 6-8 week old female CD-1 mice (Envigo) and mice for protein nanoparticle immunizations were Crl:CD1(ICR) outbred mice were purchased from Charles River (order code 022) at 7 weeks of age.
Field-collected samples	N/A
Ethics oversight	Animal experiments involving protein nanoparticles were conducted in accordance with the University of Washington's Institutional Animal Care and Use Committee (IACUC protocol 4470-01). The University of Washington's animal use program is accredited by the Association for Assessment and Accreditation of Laboratory Animal Care (Reference Assurance: #000523). Immunization studies involving lipid nanoparticles were carried out in compliance with University at Buffalo IACUC protocols (protocol BME05044Y).

Note that full information on the approval of the study protocol must also be provided in the manuscript.

Plants

Seed stocks	*Report on the source of all seed stocks or other plant material used. If applicable, state the seed stock centre and catalogue number. If plant specimens were collected from the field, describe the collection location, date and sampling procedures.*
Novel plant genotypes	*Describe the methods by which all novel plant genotypes were produced. This includes those generated by transgenic approaches, gene editing, chemical/radiation-based mutagenesis and hybridization. For transgenic lines, describe the transformation method, the number of independent lines analyzed and the generation upon which experiments were performed. For gene-edited lines, describe the editor used, the endogenous sequence targeted for editing, the targeting guide RNA sequence (if applicable) and how the editor was applied.*
Authentication	*Describe any authentication procedures for each seed stock used or novel genotype generated. Describe any experiments used to assess the effect of a mutation and, where applicable, how potential secondary effects (e.g. second site T-DNA insertions, mosiacism, off-target gene editing) were examined.*
